# Draft Genome Sequence of *Brachybacterium phenoliresistens* Strain W13A50, a Halotolerant Hydrocarbon-Degrading Bacterium

**DOI:** 10.1128/genomeA.00899-14

**Published:** 2014-09-18

**Authors:** Xinxin Wang, Zhuo Zhang, Decai Jin, Lisha Zhou, Liang Wu, Chen Li, Libin Zhao, Wei An, Yu Chen

**Affiliations:** a School of Environmental Science and Engineering, Tianjin University, Tianjin, China; b China Offshore Environmental Service Co. Ltd., Tianjin, China; c Hunan Plant Protection Institute, Changsha, China; d College of Resources and Environment, University of Chinese Academy of Sciences, Beijing, China; e Research Center for Eco-Environmental Sciences, Chinese Academy of Sciences, Beijing, China; f Shenzhen Key Laboratory of Environmental Microbial Genomics and Application, BGI, Shenzhen, China; g College of Environmental Science and Engineering, Ocean University of China, Qingdao, China; h School of Chemical Engineering and Technology, Tianjin University, Tianjin, China; i College of Biotechnology, Tianjin University of Science and Technology, Tianjin, China

## Abstract

*Brachybacterium phenoliresistens* strain W13A50 was isolated from a petroleum-contaminated saline site, which could degrade hydrocarbon under high salinity conditions. Here, we present 4.2-Mb draft genome sequence of this strain, which will provide insights into the diversity of *Brachybacterium* and the mechanism of hydrocarbon degradation in saline environments.

## GENOME ANNOUNCEMENT

Hydrocarbon-degrading bacteria in saline environment have attracted increasing attention for decades (1, 2). Recently, members in the genus *Brachybacterium* have been reported to be involved in the degradation of hydrocarbons (3, 4). However, genomic information about hydrocarbon-degrading *Brachybacterium* is still limited. Here, the draft genome sequence of halotolerant hydrocarbon-degrading *Brachybacterium phenoliresistens* strain W13A50 is presented for the first time, isolated in petroleum-contaminated saline soils from Tianjin in China.

Genomic DNA was extracted and sequenced with next generation sequencing technology by Illumina HiSeq 2000. The shotgun sequencing produced 11,562,164 paired-end reads with approximately 270-fold coverage. Filtered reads were assembled, scaffolded, and gap filled by SOAPdenovo v2.04 (5), SSPACE v2.0 (6), and GapFiller v1.10 (7). The sequences were further validated using bwa v0.7.4 (8). This assembly generated 40 contigs with an *N*_50_ length of 161,998 bp and an average length of 104,703 bp, which were assembled into 35 scaffolds with an *N*_50_ length of 218,599 bp and an average length of 119,662 bp. Genome annotation was performed by NCBI Prokaryotic Genome Automatic Annotation Pipeline (PGAAP) (http://www.ncbi.nlm.nih.gov/genome/annotation_prok/).

The draft genome composed 4.2 Mb with a G+C content of 73.2%. A total of 3,605 coding sequences (CDSs), 37 pseudogenes, 54 tRNA genes, 1 ncRNA, and 1 rRNA operon were identified. Of the CDSs, 91.0% can be assigned to clusters of orthologous groups (COGs) with carbohydrate transport and metabolism as the most abundant class, and 44.6% can be annotated into 1,232 KEGG orthologous groups by using KAAS (9), involving 198 metabolic pathways. A total of 1,146 tandem repeats were detected as revealed by Tandem Repeats Finder v4.07 (10). ISFinder revealed IS110 family dominated the insertion sequence (IS) elements (11). Two hundred seventeen potentially secreted proteins were identified by SignalP v4.0 (12). Neither clustered regularly interspaced short palindromic repeat (CRISPR) element nor prophage sequences were identified as revealed by CRISPRFinder (13) and PHAST (14). Average nucleotide identity (ANI) analysis (15) revealed that *B. phenoliresistens* W13A50 is phylogenetically related to *B. faecium* DSM4810 (76.1%) (16), *B. muris* UCD-AY4 (75.7%) (17), *B. paraconglomeratum* LC44 (76.4%) (18), and *B. squillarum* M-6-3 (76.8%) (19).

Two alkane 1-monooxygenase genes were identified, which were responsible for the degradation of alkanes. In addition, 9 genes were found to be involved in the synthesis and uptake of compatible solute, including 1 trehalose phosphatase gene, 1 maltooligosyl trehalose synthase gene, 1 trehalose synthase gene, 1 betaine-aldehyde dehydrogenase gene, and 5 glycine/betaine ABC transporter genes. These genes may be important to survival in a saline environment. Information about the genome sequence of *B. phenoliresistens* W13A50 offered an opportunity to understand the diversity of *Brachybacterium* and the mechanism of hydrocarbon degradation in saline soils.

### Nucleotide sequence accession number.

The draft genome sequence of *B. phenoliresistens* W13A50 has been deposited in GenBank under the accession no. JDYK00000000. The version described in this paper is the first version.
